# A Synthesis of Clinical Recommendations and Primary Research for Survivors of Prostate or Breast Cancer

**DOI:** 10.1188/14.CJON.667-673

**Published:** 2014-12

**Authors:** Melanie Sandoval, Jennifer Wenzel, Randy Jones

**Affiliations:** Perioperative services at the University of Colorado Hospital in Aurora; Department of Acute and Chronic Care at Johns Hopkins University in Baltimore, MD; Research in the School of Nursing at the University of Virginia in Charlottesville

**Keywords:** oncology, prostate cancer, breast cancer, cancer treatment

## Abstract

Studies have documented the efficacy of cancer treatments available, specifically for patients with prostate or breast cancer, but few articles have compared prostate or breast cancer recommendations from the patient’s perspective. In this article, the authors compare and contrast published clinical recommendations for advanced practice RNs who treat patients with prostate or breast cancer to qualitative studies that explore the experiences of cancer survivors. Treatment options, along with recommendations and resources, are included. The nurse clinician’s role in caring for patients with prostate or breast cancer is diverse and complex, and evidence supports the role of the nurse clinician in improving patient care. Implementing findings from qualitative studies that focus on patients’ perspectives in conjunction with clinical recommendations is essential when developing care plans for patients with cancer.

The advanced practice RN (APRN) provides care to a number of patients who vary in age and income, as well as in disease type and chronicity. Just as health care continues to increase in complexity, so does the role of the APRN. Therefore, the APRN must acquire a deeper, broader range of specialty skills, knowledge, and resources to effectively manage the care of this growing population. Given the aging general population and the overall increase in patients diagnosed with cancer, the APRN likely will be managing the care of cancer survivors or assuming the role of the primary care provider for some patients. The APRN is being challenged to develop the skills and knowledge necessary not only to manage the medical care of cancer survivors but also to help patients, families, and caregivers address spiritual, financial, emotional, and personal burdens.

The Institute of Medicine (IOM) has outlined the needs of patients with cancer and their families, including unmet psychosocial needs (Adler & Page, 2008). To satisfactorily meet the IOM’s recommendations and to effectively manage the care of complex and chronically ill individuals, the APRN must synthesize and apply knowledge and evidence from clinical practice, as well as from published research and clinical guidelines that focus on screening, diagnosis, treatment, and follow-up, to provide care that meets the unique needs and preferences of each patient with cancer.

Most APRNs access and apply clinical guidelines to manage the care of patients with cancer. Although guidelines are often evidence based, they may not address the personal preferences of the patient. The financial resources of the patient may not be fully discussed during treatment planning. For example, the cost of a magnetic resonance imaging scan may not be evaluated prior to diagnostic ordering. Cost-effective measures based on the patient’s financial resources may not be fully explored, and alternative measures may not be considered. In addition, certain patients may choose to pursue aggressive treatments for their cancer, whereas others, based on personal beliefs, may choose more conservative treatments. The beliefs and resources of the patient should be explored during treatment planning.

Clinical guidelines are accessible and useful to nurse clinicians in inpatient and outpatient facilities. Unfortunately, few guidelines reflect patients’ experiences. Limited information exists to help guide the care of cancer survivors from underserved and underprivileged backgrounds, many of whom may experience stressors in addition to a cancer diagnosis. For example, African American men aged 65 years or older are disproportionately affected by cancer. At the time of their initial diagnosis, these individuals often present with an advanced stage of cancer and require a greater amount of resources to effectively treat the cancer because of the late stage of the disease and their age (Jones et al., 2011). The treatment options offered to those individuals, therefore, may be more costly. According to Jones et al. (2011), the beliefs, values, and financial resources of patients should be considered in the context of their disease. Suggesting that a 70-year-old African American man with end-stage metastatic prostate cancer undergo costly, highly focused radiation treatments that have serious, unpleasant side effects may not enhance his overall quality of life. The APRN should closely assess the individual patient’s needs, beliefs, and resources to develop an effective plan of care.

Clinical recommendations may be helpful to the APRN in terms of a patient’s initial care, but they are limited in their ability to guide the APRN in developing a plan that includes the patient’s preference. In a study by Jones et al. (2011), a group of African American prostate cancer survivors and a group of African American breast cancer survivors described their illness experiences, including their difficulties and triumphs. Descriptive narratives, combined with clinical guidelines, may provide the APRN with the fundamental elements needed to develop an effective, holistic, and individualized plan of care for a patient with cancer.

Unfortunately, few studies have explored the perceptions of patients before, during, or after treatment for prostate or breast cancer, although treatment may greatly affect quality of life (Northouse, Katapodi, Song, Zhang, & Mood, 2010). Birnie and Robinson (2010) presented five main treatment options offered to patients diagnosed with localized prostate cancer: active surveillance, radical prostatectomy, external beam radiotherapy, brachytherapy, and cryotherapy. These treatments vary greatly, and their effects on the patient’s overall functioning may differ. Likewise, treatment for early-stage breast cancer may consist of surgery with or without radiation, chemotherapy, or hormone therapy. Few studies have synthesized original research data that reflects the challenges and preferences of treatment as described by the patient.

This synthesis of original research studies describing the experiences of prostate or breast cancer survivors was designed with the goal of providing insights from patients’ perspectives to enhance the APRN’s understanding of the specific challenges facing patients with cancer. The purpose of this review is to outline actual patient experiences, which can help APRNs increase the quality of care and treatment options.

## Methods

This review of literature focused on locating, summarizing, and synthesizing clinical studies that outlined treatment options and recommendations for providers caring for patients with a current or past diagnosis of prostate or breast cancer. The authors selected six clinical studies that addressed clinical treatment options for prostate or breast cancer, omitting studies that focused on theoretical models to treat patients with cancer if they were not specific to prostate or breast cancer. Those recommendations were then compared to five qualitative studies that explored the experiences of prostate or breast cancer survivors, including their perceptions of cancer treatments and any related barriers or facilitators to treatment. Articles that reanalyzed original studies were not included. Clinical studies conducted from 2006–2011 were reviewed, and CINAHL® and PubMed databases were used to search for articles that included clinical recommendations and perceptions of patients with a current or past diagnosis of prostate or breast cancer. The following Medical Subject Headings terms were included: APN recommendations for prostate cancer, APN recommendations for breast cancer, prostate cancer, breast cancer, treatment, patient treatment, and *patient values*.

### Inclusion Criteria

Inclusion criteria for the review consisted of an article’s (a) being an original research study, (b) featuring a sample in which the patients had a current or past diagnosis of prostate or breast cancer, and (c) having a publication date of 2006 or more recent and being written in English. Studies that focused on pharmacologic interventions were excluded because they were not applicable to the aim of the review. The authors reviewed and synthesized the clinical recommendations from the articles written for APRNs managing the care of patients with prostate or breast cancer diagnoses, and they also conducted a secondary analysis of data from narratives taken from participant interviews. This information, along with specific recommendations for the APRN, is summarized in Table 1; those recommendations are aligned with the components that should be included in a survivorship action plan as recommended by the Centers for Disease Control and Prevention (2004). The interviews were conducted as part of five qualitative studies describing the challenges and experiences of older African American men diagnosed with prostate cancer and African American women diagnosed with breast cancer. The authors’ analysis included obtaining and reviewing the original transcripts of participant narratives from the five studies.

The primary studies included in this synthesis were guided by phenomenologic qualitative methodology (Cohen, Kahn, & Steeves, 2000). These studies involved semistructured interviews with participants who had been diagnosed with prostate or breast cancer. The phenomenologic methodology used in the primary research was based on the concept that meaning is derived by participants through a process of narrative construction. The methods of analysis are clearly described by the investigators in each of the respective qualitative studies (Jones et al., 2011; Klimmek, Snow, & Wenzel, 2010).

## Results

The authors’ review of recently published guidelines for APRNs who treat patients with prostate or breast cancer confirmed that guidelines for survivors of cancer in regard to cancer screening, complications, and recommendations for lifestyle modifications are readily available. Meta-analyses regarding treatment-related mortality among patients with prostate or breast cancer are available for review (Newman et al., 2006). Randomized, controlled trials to determine the overall risk of fatal adverse events associated with different treatment modalities are numerous but do not often include the perspective of the patient. However, many do include physical side effects related to treatment. These studies are often large and offer detailed statistical analyses, yet little patient perspective is available to a nurse clinician. For example, Ranpura, Hapani, and Wu (2011) synthesized data examining treatment-related mortality among 10,217 patients with cancer. Although few articles outlined the negative impact that treatment may have on a patient’s quality of life, Carter, Bryant-Lukosius, DiCenso, Blythe, and Neville (2011) explored the supportive care needs of men in Canada with advanced prostate cancer (N = 29). The researchers concluded that nurses play an important role in addressing the needs of patients; they are fundamental in providing emotional support and the information necessary to cope with physical problems related to treatment (i.e., sexual and urinary function). A nurse clinician’s increased understanding of what type of emotional and social support patients need, along with a willingness to provide guidance for treatment, may optimize patients’ overall quality of life.

Studies (Kim, 2007; Klimmek et al., 2010; Wenzel, Jones, Klimmek, Krumm, et al., 2012; Wenzel, Jones, Klimmek, Szanton, & Krumm, 2012) have documented the burden of financial stress on the cancer survivor and have also examined the association between high financial burden and decreased chance of cancer survival (Ward et al., 2008). However, few of these studies have outlined resources that APRNs may use to assist patients who experience financial challenges.

Using data from semistructured interviews with patients undergoing cancer diagnosis and treatment, Klimmek et al. (2010) described the challenges and stress experienced by patients with breast cancer who were dealing with managed care organizations (MCOs) during treatment and early follow-up. The challenges were grouped into five MCO-related tasks: (a) interacting with MCOs, (b) understanding written information from MCOs, (c) obtaining authorizations, (d) paying bills and planning for the costs of care, and (e) obtaining assistance with MCO-related tasks (Klimmek et al., 2010). The recommendations for nursing assessment and intervention were targeted toward an audience of oncology nurse clinicians, but they also contained essential information for practicing APRNs who care for patients with cancer. For example, Klimmek et al. (2010) found that patients had trouble understanding written information from MCOs. This challenge emphasizes the need for practitioners to spend extra time with each patient and his or her family to explain complex terminology and to be available to provide answers for questions that may arise. In fact, the findings of Klimmek et al. (2010) suggest that the APRN may need to include a community support person or community health worker (CHW) as part of the treatment team to facilitate navigation of the insurance and healthcare systems. A community support person can also assist patients with tasks such as driving to appointments, whereas a CHW can assist with their health-related needs, including administering medication.

### Themes

Jones et al. (2011) described the experiences of urban and rural African Americans who had undergone prostate cancer treatment and reported findings similar to those in the breast cancer study by Klimmek et al. (2010). Four themes emerged in the study by Jones et al. (2011): (a) the need for greater health and cancer-specific education, (b) the importance of faith and spirituality, (c) the availability of support, and (d) the difficulty with identifying and articulating financial needs. Jones et al. (2011) emphasized the need for additional support of patients with prostate cancer to facilitate learning about healthcare preservation and management and healthcare navigation.

**FIGURE 1:**
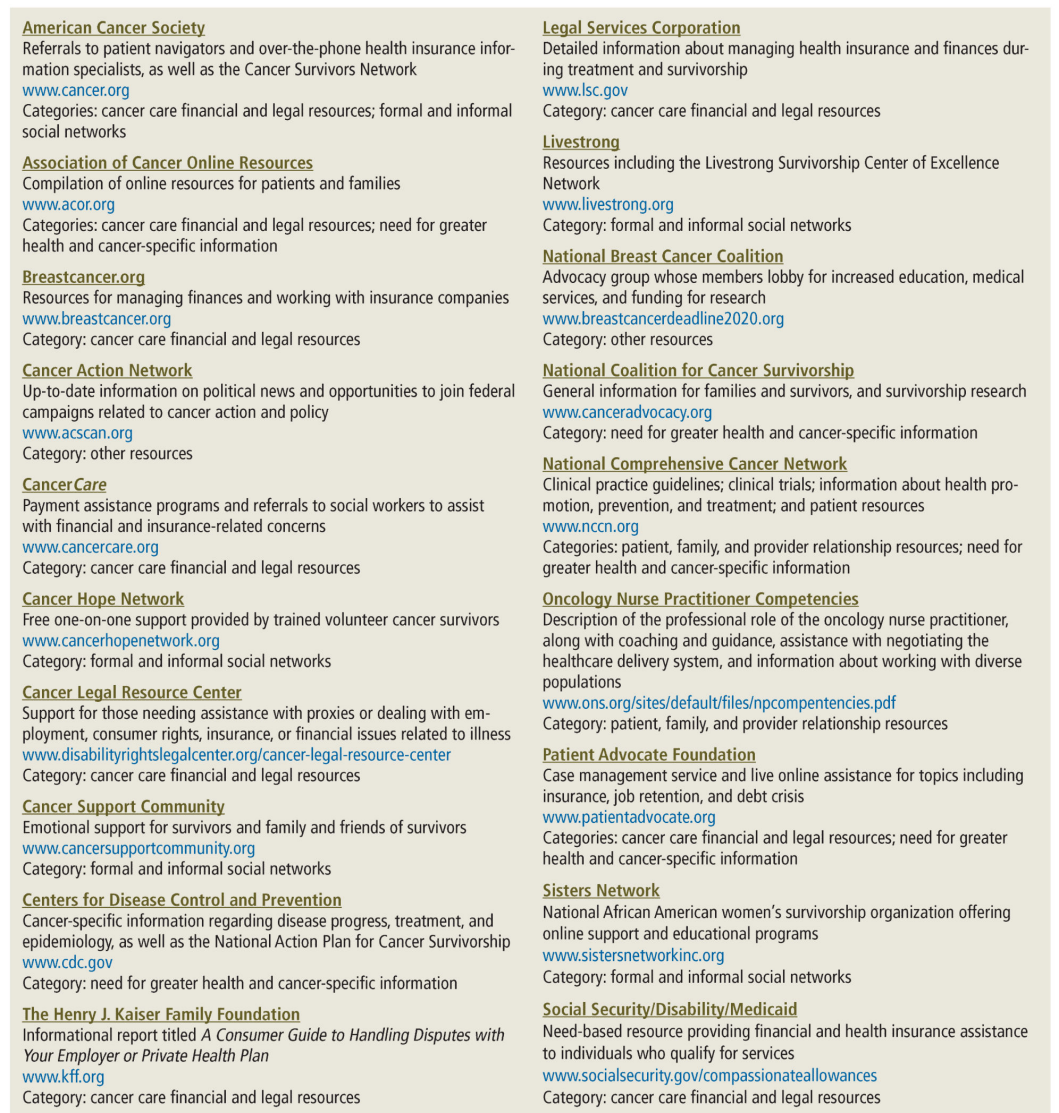
Online Resources for Patients With Prostate or Breast Cancer

The overarching theme captured in both studies was cancer survivors’ need for increased support to enhance the continuity of care and to decrease the overall burden of the cancer diagnosis. Themes that emerged also highlighted the preferences and needs of African American cancer survivors and their family members. Figure 1 presents a list of online resources with information relevant to nurse clinicians and other providers of care who work with patients diagnosed with prostate or breast cancer. Websites that address the financial and legal implications of cancer costs and insurance burdens are also included. These resources are organized into five categories: (a) patient, family, and provider relationship resources, (b) cancer care financial and legal resources, (c) need for greater health and cancer-specific information, (d) formal and informal social networks, and (e) other resources.

## Discussion

Many of the reviewed studies included recommendations for the treatment of prostate and breast cancer. The current review compares clinical recommendations to findings from five qualitative studies that explored the values and beliefs of patients who had received a diagnosis of prostate or breast cancer. By outlining patients’ perspectives in regard to what treatment should include and revealing the challenges that patients face, this review also highlights the importance of the nurse’s role in treatment.

Few studies provided clinical recommendations based on diagnosis, treatment, patient preferences, and patient resources. A treatment plan should be based on the prognosis of the disease and the values and resources of the patient. The resources available to each patient vary, and the APRN should explore them prior to making treatment recommendations or developing a plan of care for the patient. APRNs should collaborate with the treatment team, the patient, and the patient’s family and friends to create a plan of care that is suitable for each patient. To achieve optimal results, the nurse clinician and the APRN must be actively involved in the patient’s care.

### Limitations

This review has several limitations, as does the comparison of clinical recommendations and qualitative research. First, the patient populations in the clinical studies were not well described and may not reflect beliefs and values similar to participants in the research-based study. A limited number of clinical trials focused solely on African Americans’ perspectives related to cancer treatments; therefore, a need exists for further clinical research with similar populations of patients. Those findings can then be compared to qualitative studies with African American participants. In addition, the number of participants in the qualitative study was limited. Future studies that focus on the treatment experiences of patients with cancer should have a greater number of participants in multiple geographic settings. Doing so would strengthen the findings for a more generalizable representation.

## Implications for Nursing

In a time of increasing patient workloads and ongoing plans for healthcare reform in the United States, APRNs may have increasing difficulty committing the necessary time to exploring complex cancer-related healthcare needs with patients. Cancer survivors, who are often living with other chronic diseases, require extra support and resources. Certain individuals face additional barriers, such as limited education and finances, along with an overall lack of trust in healthcare providers—all of which may further delay or hinder treatment (Jones, Steeves, & Williams, 2009a, 2009b). Understanding the complexities and concerns of individuals who are diagnosed with cancer can prepare APRNs to effectively address complex issues faced by patients. A more in-depth understanding will allow APRNs to use their time and energy more efficiently while decreasing organizational and patient costs.

From the authors’ review of recently published studies that have focused on patients’ perceptions of challenges associated with cancer diagnosis, the role of the APRN goes far beyond prevention, screening, and treatment, and it should include preparing a customized plan of care for each patient in conjunction with evidence-based clinical guidelines. In addition, the support resources in this article can be used to provide patient education regarding financial concerns and overall care.

## Figures and Tables

**TABLE 1:** Perceived Needs, Challenges, and Clinical Recommendations for Patients, Families, and Clinicians

Perceived Needs and Challenges	Narrative	Narrative Source	Recommendations
Need for a trusting relationship with healthcare provider	“I was not really thinking about prostate cancer screening. My relatives had prostate cancer. I am going forward with it. After talking to my doctor, he encouraged it, and I went to the doctor.”	Jones et al., 2009a	Encourage patients to return for follow-up appointments. Actively listen to the concerns voiced by patients and family members, and remain available to support patients and provide healthcare information.
Difficulty articulating financial needs	“No, Humana. So now I’m using Humana, but … they take Medicare. I don’t know exactly how to explain it. I don’t feel like I’m getting [any] benefit out of it.”	Wenzel, Jones, Klimmek, Krumm, et al., 2012	Schedule new patients with extra time to familiarize them with the process of contacting MCOs, or designate office personnel to assist with new patients. Consider developing a questionnaire that asks what challenges patients have experienced and track these forms in a database that can also be used to document the invaluable role of the APRN. Provide office personnel with a “cheat sheet” based on common challenges that patients identified in their interactions with MCOs.
Difficulty understanding the language used in written correspondence with MCOs	“No, because I don’t know who to talk to. That’s what I’m trying to find out now, so I can talk to someone that would help me, you know.”	Klimmek et al., 2010	Encourage patients to bring in letters from MCOs during scheduled office visits so they can receive assistance with understanding complex information and difficult terminology. Consider asking a nurse leader to designate certain hours one day a week to provide patients with additional information or clarification of difficult written information.
Difficulty obtaining authorization from MCOs for diagnostics, specialists, referrals, and non- formulary medication, and a lack of transparency in the authorization process	“I put in for the VA, but I was denied…. They said I didn’t qualify financially, and, the other thing, I didn’t have any service to connect the disabilities.”	Klimmek et al., 2010	Discuss and offer alternative diagnostics or treatments, explaining cost, benefits, and risks to patients. Include costs covered by insurance and costs for which the patient would be responsible. Rather than routinely offering the latest or usual form of care, spend time with patients and family members to determine personal preferences and needs, including homeopathic treatments.
Higher co-payments for cancer providers and medications to treat cancer or the side effects of treatment	“The first time I went through all of this experience of cancer, we had insurance that was $15 co-pay and they [took] care of the rest, and when I retired, it was a different story. It was a different story.”	Klimmek et al., 2010	Become familiar with and educate all office and medical personnel about the American Cancer Society’s costs of cancer care document. Provide patients with information on the topic of expected side effects of cancer treatments and nonpharmacologic interventions while patients are waiting to be seen, giving them a chance to read the material and ask questions. Collaborate with specialists to develop a plan of care in which the APRN can prescribe and order certain treatments to alleviate costs.
Unexplained fees related to cancer treatment and frustration with billing errors	“I was doing really well before I turned 65. When I turned 65 … they dropped the ball on everything: ‘You got to pay for this, you got to pay for that.’ My medicine went up.”	Klimmek et al., 2010	Provide all patients with a referral to the account manager and billing office specialist. Refer them to appropriate legal websites.
Psychosocial problems associated with cancer diagnosis	“[There] used to be a time I had to go to the bathroom so many times a night … about four or five times a night, sometimes more than that.” [man with prostate cancer] “All I wanted to do was lay down, lay down, lay down. Forget about the food—just lay down. It went on for a long time.” [woman with breast cancer]	Jones et al., 2009a	Provide appropriate counselor and group referrals. Consider creating a holistic treatment plan that is individually tailored to meet the needs of each patient. Include a community support person in the planning.
Geographic location	“The only problem … was transportation. See, Medicare wouldn’t pay for my transportation. Because, see, I was under Medicare, not Medicaid. Medicare won’t pay for transportation.”	Jones et al., 2011	Determine the geographic location in which the patient resides. Discuss the availability of transportation services, clinics, pharmacies, and CHWs to alleviate the burden of getting to appointments and picking up medications.
Need for greater health and cancer-specific education	“We really don’t get told the truth, exactly what’s what…. I think it’s … just one of the things that we don’t get a real true [diagnosis] and be told the truth in all cases.” “Maybe even though there are options, and, of course, you, as the patient, have the ultimate decision-making ability, but people may steer you in certain directions.”	Jones et al., 2009a	Mentor clinical nurses in the workplace as educational group leaders. Offer a variety of seminars on topics such as nutrition, identifying secondary illnesses not related to the primary cancer, and secondary cancers associated with specific cancers (including prevention, along with signs and symptoms). Implement a model that includes the community support person for the patient in the education.
Need for spiritual support	“The church, they brought me money; they brought all types of goodies; they never let you know that they’re not there.”	Jones et al., 2009a	Offer spiritual services to outpatients when they present for office visits or by appointment, as opposed to only during inpatient treatment. Consider designating a room as a spiritual or meditation area for patients and community support people.
Lack of available support	“I have two sister-in-laws that came right regular, and my nieces, they came and cleaned the house for me; that was really beautiful. They did a lot for me.”	Jones et al., 2011	Embrace a model of collaboration that includes specialists, social workers, financial personnel, family members, community support people, and CHWs.
Assistance identifying and articulating financial needs	“I know this man used to live not too far from me. These people would come and clean his house and make him make sure he took a bath; they were all men. They would come up—a group of men would come out to his house, clean his house, and straighten it up, and, you know, keep his kitchen straight, and cook him a meal, and make sure he [took] a bath, but I don’t know whether they were called that or not. A group of people used to come to his house.”	Klimmek et al., 2010	Encourage the CHW or community support person to attend office visits, procedures, or treatments with the patient and to prepare notes or questions prior to the appointment.
Need for a social support person in the community to assist with basic needs	“The technicians, when I went down there and the music is playing in there and everything, and they said, ‘Do you like the music playing?’ I said, ‘Not really.’ They said, ‘Well, you can bring some of your own.’ I brought some CDs in there, and they started playing them, and they liked them.”	Jones et al., 2011	Assess the patient’s support system prior to discharge or during initial visit.
Need to address economic disadvantages	“My children [were] there for me because they had to buy them high-priced pills. I didn’t have no money to get them with. I didn’t know how I was going to make it, but they were there, and they’d bring money from here and there or whatever, trying to help me. They’re still trying to help me with that bill.”	Jones et al., 2011	Provide patients with a list of Internet sites and phone numbers related to legal or financial information. Consider having a computer that can be used by patients. Provide patients with time slots to use the computer to increase independence and to access resources that address specific concerns. Network with social workers and agencies that offer financial support to patients in need.
Internal and community resources recognized as a source of strength	“I think the first important person to me was my wife because we sat down and discussed it. And equally important was the doctor. Dr. — was very, very encouraging from day one [because of] his explanation about the treatment.”	Jones et al., 2011	Familiarize yourself and the treatment team with available CHW resources and other resources available to the patient at home or in the community, and make contact with the agency or relevant person prior to the patient’s departure.

APRN—advanced practice RN; CHW—community health worker; MCO—managed care organization

## Implications for Practice

▶ Increase understanding of the unique needs of prostate or breast cancer survivors.
▶ Take time to assess patients’ financial resources.
▶ Discuss the risks and benefits of available treatments in the context of each patient’s beliefs and values.
